# Heme Drives Susceptibility of Glomerular Endothelium to Complement Overactivation Due to Inefficient Upregulation of Heme Oxygenase-1

**DOI:** 10.3389/fimmu.2018.03008

**Published:** 2018-12-20

**Authors:** Olivia May, Nicolas S. Merle, Anne Grunenwald, Viviane Gnemmi, Juliette Leon, Cloé Payet, Tania Robe-Rybkine, Romain Paule, Florian Delguste, Simon C. Satchell, Peter W. Mathieson, Marc Hazzan, Eric Boulanger, Jordan D. Dimitrov, Veronique Fremeaux-Bacchi, Marie Frimat, Lubka T. Roumenina

**Affiliations:** ^1^INSERM, UMR_S 1138, Centre de Recherche des Cordeliers, Paris, France; ^2^INSERM, UMR 995, Lille, France; ^3^University of Lille, CHU Lille, Nephrology Department, Lille, France; ^4^Sorbonne Universités, UPMC Univ Paris 06, Paris, France; ^5^Université Paris Descartes, Sorbonne Paris Cité, Paris, France; ^6^University of Lille, INSERM, CHU Lille, Department of Pathology, UMR-S 1172 - Jean-Pierre Aubert Research Center, Lille, France; ^7^Bristol Renal, University of Bristol, Bristol, United Kingdom; ^8^University Lodge, University of Hong Kong, Hong Kong, Hong Kong; ^9^Assistance Publique – Hôpitaux de Paris, Service d'Immunologie Biologique, Hôpital Européen Georges Pompidou, Paris, France

**Keywords:** atypical hemolytic uremic syndrome, complement system, endothelial cells, heme, heme oxygenase-1, thrombomodulin

## Abstract

Atypical hemolytic uremic syndrome (aHUS) is a severe disease characterized by microvascular endothelial cell (EC) lesions leading to thrombi formation, mechanical hemolysis and organ failure, predominantly renal. Complement system overactivation is a hallmark of aHUS. To investigate this selective susceptibility of the microvascular renal endothelium to complement attack and thrombotic microangiopathic lesions, we compared complement and cyto-protection markers on EC, from different vascular beds, in *in vitro* and *in vivo* models as well as in patients. No difference was observed for complement deposits or expression of complement and coagulation regulators between macrovascular and microvascular EC, either at resting state or after inflammatory challenge. After prolonged exposure to hemolysis-derived heme, higher C3 deposits were found on glomerular EC, *in vitro* and *in vivo*, compared with other EC in culture and in mice organs (liver, skin, brain, lungs and heart). This could be explained by a reduced complement regulation capacity due to weaker binding of Factor H and inefficient upregulation of thrombomodulin (TM). Microvascular EC also failed to upregulate the cytoprotective heme-degrading enzyme heme-oxygenase 1 (HO-1), normally induced by hemolysis products. Only HUVEC (Human Umbilical Vein EC) developed adaptation to heme, which was lost after inhibition of HO-1 activity. Interestingly, the expression of KLF2 and KLF4—known transcription factors of TM, also described as possible transcription modulators of HO-1- was weaker in micro than macrovascular EC under hemolytic conditions. Our results show that the microvascular EC, and especially glomerular EC, fail to adapt to the stress imposed by hemolysis and acquire a pro-coagulant and complement-activating phenotype. Together, these findings indicate that the vulnerability of glomerular EC to hemolysis is a key factor in aHUS, amplifying complement overactivation and thrombotic microangiopathic lesions.

## Introduction

The atypical hemolytic uremic syndrome (aHUS) is a rare, kidney-predominant, thrombotic microangiopathy (TMA) associated with the formation of fibrin-platelet clots in microvessels which trigger mechanical hemolysis (1). A dysregulated complement alternative pathway (AP) plays a key role in aHUS, as suggested by genetic abnormalities in AP proteins found in up to 60% of patients (2, 3) and the efficacy of anti-C5 therapy (4). Although the complement overactivation is systemic, a particular renal tropism of the TMA lesions exists, and injury of others organs is unusual in aHUS (5–10% of patients) (2, 5).

This susceptibility of renal endothelial cells (EC) to complement overactivation remains poorly understood. Despite the morphologic and functional differences between glomerular EC and other microvascular EC, no major differences were observed in the basal expression of complement regulators or complement component C3 deposits in resting state (6–10). The high expression of complement proteins in kidneys could explain the greater susceptibility of glomerular EC to complement attack. The EC-derived C3 and Factor B are, however, present at lower concentrations in kidney compared to the liver-derived C3 and FB in blood, to which endothelium is exposed *in vivo*. Additional factors are necessary to fully explain the vulnerability of the glomerular endothelium to complement-mediated injury and TMA in aHUS.

Cell-free hemoglobin and heme, released during hemolysis, are potent promoters of pro-inflammatory and pro-oxidant effects (11–13) and can induce EC phenotypical changes, affording them complement activating properties (8, 14, 15). Under hemolytic conditions EC also up-regulate the cytoprotective heme-degrading enzyme heme oxygenase 1 (HO-1) (15), and this overexpression was associated with increased resistance to complement activation in HUVEC (16). Nevertheless, the implication of HO-1 in the protection of glomerular endothelium has not, to our knowledge, been studied.

We here show that endothelial heterogeneity is apparent under hemolytic conditions: the microvascular EC, and particularly the glomerular EC, become vulnerable to injury through differences in their C3 regulation and heme degradation compared with macrovascular EC. These results might explain, at least in part, the renal tropism of complement-mediated TMA lesions in aHUS.

## Materials and Methods

### Reagents

The oxidized form of heme (hemin, ferriprotoporphyrin IX), designated as heme, (Sigma or Frontier Scientific) and Sn(IV) mesoporphyrin IX (SnMPIX) (Frontier Scientific) were suspended at 10 mM in 50 mM NaOH, 145 mM NaCl, and therafter diluted in the appropriate vehicle (medium for culture cells, PBS for animal experimentation).

### Animal Experimentation

All experiments were conducted in accordance with the recommendations for care and use of laboratory animals of the French Ministry of Agriculture and with the approval of the Charles Darwin Ethics Committee for animal experimentation (Paris, France) number APAFIS#3764-201601121739330v3. Six to eight-week-old female C57Bl/6 mice from Charles River Laboratories (L'Arbresle, France) were treated with intraperitoneal injection of 200 μL phosphate buffer saline (PBS, Dulbeco) or freshly prepared heme (40 μmol/kg corresponding to 27 μg/g body weight) at day 0 and 1. At day 2 mice were culled and the organs were then recovered: kidneys, lungs, heart and skin (1 cm2) were preserved directly in plastic molds containing optimal cutting temperature (OCT) compound, placed on dry ice and frozen. Brains were otherwise frozen in isopentane at −70°C to preserve the tissue architecture before being placed in OCT and frozen.

### Immunohistochemistry (IHC) and Immunofluorescence (IF)

Frozen organs sections (6 μm) were fixed in acetone. The primary antibodies were: Heme Oxygenase-1 (HO-1; rabbit anti-mouse, Abcam Ab13243, 5 μg/ml), C3b/iC3b (rat anti-mouse, Hycult biotech, HM1065, 1 μg/ml), Thrombomodulin (TM; rabbit anti-mouse, R&D systems, MAB3894, 20 μg/ml), CD31 (rat anti-mouse, Abcam Ab7388, 2 μg/mL), von Willebrand Factor (vWF; sheep anti-mouse, Abcam, Ab11713, 1/100). Staining was revealed by goat anti-rabbit AF647 (Thermoscientific, A21244, 5 μg/mL) or goat anti-rat AF555 (Thermoscientific, A21434, 5 μg/mL). Slides were scanned by Nanozoomer (Hamamatsu) and AxioScan (Zeiss) for IHC and IF, respectively.

### Endothelial Cells Assays

#### Cells Culture

Human umbilical vein endothelial cells (HUVEC—a model of macrovascular EC), a human dermal EC line (HMEC—a microvascular EC model), conditionally immortalized (GENC) and human primary (HRGEC) glomerular EC were compared. HUVEC (*n* = 7 donors) were obtained from CHU Lille (France), HMEC were from ATCC (17) (US), GENC were from Dr. Satchell (Bristol, UK) (18) and HRGEC (*n* = 3) were from iCelltis (Toulouse, France). Cells were cultured as previously described (9, 10, 18): briefly, the growth medium was M199 20% fetal calf serum and 20% EGM2-MV (Lonza) for HUVEC, and EGM2-MV for the other cell types. HUVEC and HRGEC were used for experiments until passage 4, HMEC between passages 2–7 and GENC between passages 23 and 30. Cells were exposed to heme at indicated doses overnight or for 30 min in serum-free medium OPTI (Thermo Fisher), or overnight to the pro-inflammatory cytokines TNFα and IFNγ (PeproTech) at 10 ng/ml and 10^3^ U/ml in complete medium, respectively. Alternatively, HUVEC were exposed to heme or SnMPIX overnight before being re-challenged, or not, with 50 μM of heme. Dead cells were removed by washing. Normal human serum (NHS) was used as a source of complement.

#### Flow Cytometry

Cells were washed, detached, labeled and analyzed by flow cytometry (BD LSR II), and the data assessed using FCS Express software (*De Novo* software). Antibodies were diluted in PBA (PBS, BSA 0.5%, Azide 0.1%): anti-C3c (Quidel, A205, 10 μg/ml), anti-FH (antiFH#1, Quidel, A229, 55 μg/ml), anti-MCP-PE (Bio Rad, MCA2113, 10 μg/ml), anti-DAF (Bio Rad, MCA1614, 10 μg/ml). Staining was revealed by goat anti-mouse IgG PE (Beckman Coulter, IM0551). Cell viability was followed by annexin V-APC/DAPI staining (BD Bioscience).

#### RTqPCR

RNA extraction from cells was performed with a Qiagen kit. Quality and quantity of RNA were measured by an Agilent 2100 Bioanalyzer (Agilent Techonologies). RNA Integrity Number was considered acceptable if >9. After standard RT-PCR, amplification of cDNA was proceeded with following probes: actin-4332645, TM-hs00264920-s1, HO-1-hs01110250_m1, KLF2-Hs00360439_g1, KLF4-Hs00358836_m1, NRF2-Hs00975961_g1, BACH1-Hs00230917_m1. Data were analyzed by SDS2.3 and RQ manager software. The mean cycle threshold (CT) values for both the target and internal control (β*-actin*) were determined for each sample. The fold change in the target gene, normalized to β*-actin* and relative to the expression of untreated HUVEC, was calculated as 2–ΔΔCT (19).

#### Western Blot (WB)

Cells were lysed in RadioImmunoPrecipitation Assay (RIPA) buffer and deposited at 10 μg/ml on 10% pre-casted gels (Life Technology). After transfer, the HO-1 and actin were probed by a rat anti-human HO-1 IgG2b (R&D systems, MAB3776, 2 μg/ml) and a rabbit anti-actin (Sigma Aldrich, A2066, 1/10000). Secondary antibodies were goat anti-rat IgG-HRP (R&D systems, HAF005, 1/5000) and goat anti-rabbit IgG-HRP (Santa Cruz Technology, sc-2004, 1/10000). Blots were revealed by chemiluminescence (Super Signal West, Extended Duration Substrate, Thermoscientific myECL).

### Study of C3b Cleavage

To study the contribution of TM to the cleavage of C3b by Factor I (FI) in the presence of Factor H (FH) as a cofactor, purified C3b (Calbiochem), diluted in Tris buffer (10 mM, NaCl 40 mM, pH7.4) at 20 μg/ml (300 μl), was incubated with human albumin (CSL Behring, 10 μg/ml), TM (R&D Systems, 10 μg/ml, 10 μl), or without protein for 10 min on ice. FH (20 μg/ml, 10 μl) was added on ice for 10 min. Each sample was separated into 4 tubes, and 4 μl of FI (10 μg/ml) was added per tube. DTT-Blue Reducing sample buffer (13 μL) was added at *t* = 0, 30 s, 2 min or 10 min to stop cleavage of C3b. Samples were denatured, resolved on a 10% gel and transferred onto a nitrocellulose membrane. The antibodies used for visualization by WB were an anti-C3 polyclonal goat antiserum (Merck Millipore, 204869, 1/5000) and a goat anti-rabbit antibody IgG-HRP (Santa Cruz, sc-2004, 1/5000).

### Patients' Kidney Biopsies

Biopsies from two aHUS patients - P1 carrying C3 p.R161W [C3 gain of function (9)] and P2, having homozygous FH mutation p.C564F (FH deficiency)—were retrieved from the archive of the Pathology Institute of CHU Lille, France. P1 had a hemolytic anemia and histological analysis described typical lesions of glomerular and arteriolar TMA. Hemolysis was corrected at the moment of P2 biopsy, which reported predominant chronic TMA lesions, characterized by vessel wall thickening and thrombotic occlusion of many arterioles. Perls' Prussian blue staining identified hemosiderin deposits. Immunohistochemical analysis for HO-1 (rabbit anti-HO-1, Abcam) was performed on deparaffinized slides using Ventana XT autostainer (Ventana Medical systems). Immunofluorescence with rabbit anti-C3c (Dako) was performed on frozen slides. Secondary antibodies were coupled to Fluorescein IsoThioCyanate—FITC (Sigma Aldrich). A normal protocol, kidney allograft biopsy performed 3 months after transplant was used as a negative control and biopsies of two patients with chronic hemolysis (hemolysis associated with prosthetic heart valve) were used as positive controls for hemosiderin detection and tubular staining of HO-1 (20). Whole slides were scanned or analyzed by an Olympus microscope (Life Sciences Solutions) or Nanozoomer (Hamamatsu).

### Statistics

Statistical analysis was performed with R software (21). The ggplot2 package (22) was used for graphical representations. Data are presented as medians and interquartile ranges. After a Kruskal-Wallis test, a Dunn's test was used for multiple pairwise comparisons. A *p*-value < 0.05 was considered significant.

## Results

### Heme-Induced C3 Fragment Deposition With a Particular Renal Tropism *in vivo*

**Figure 1:**
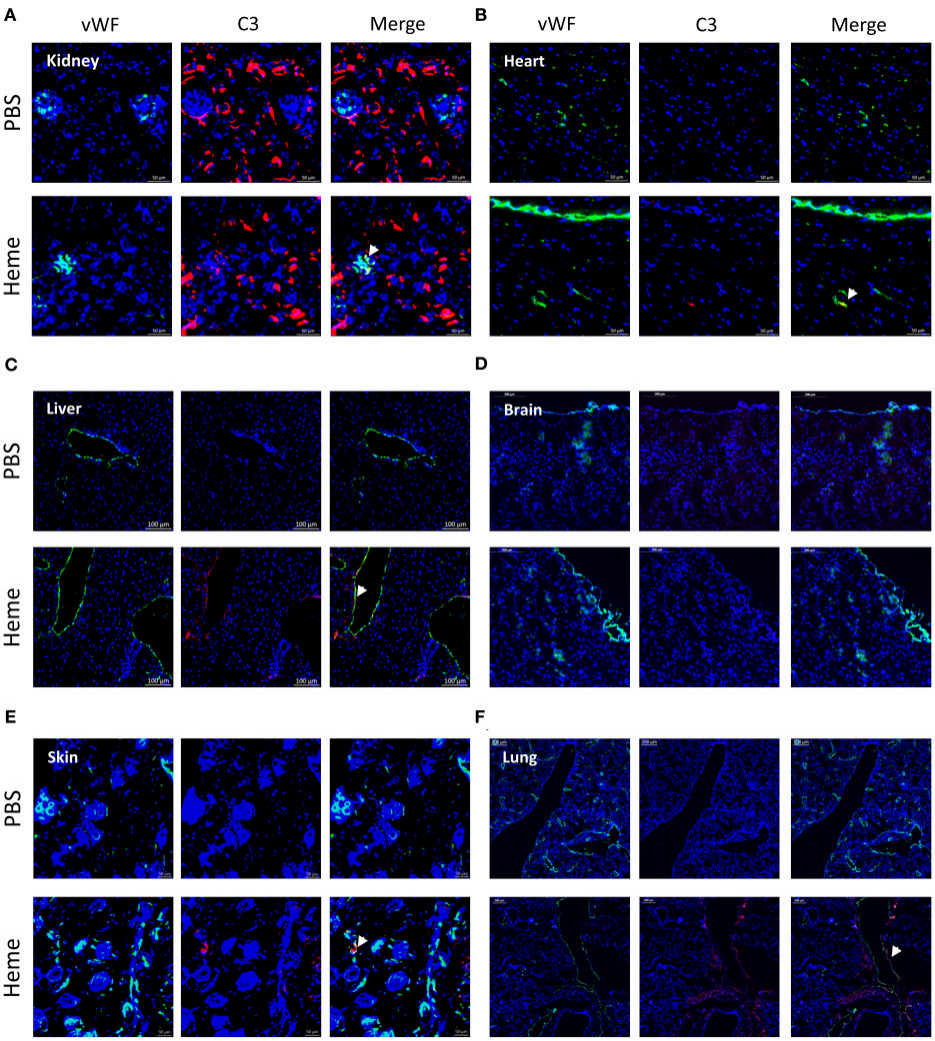
Heme-induced complement C3 fragment deposition with a particular renal tropism *in vivo*. **(A-F)** C3b/iC3b staining (red), wWF staining (green) and colocalization vWF—C3b of frozen kidney x15 **(A)**, heart x15 **(B)**, liver x8 **(C)** brain x8 **(D)**, skin x15 **(E)**, and lung x5 **(F)**; sections of mice treated with PBS (negative control, upper panel) or heme (lower panel) studied by immunofluorescence. Representative images of 3 independent experiments with 3 or 5 mice per group.

We performed a comparative analysis of the deposition of C3 activation fragments in mouse organs under control conditions (injection of PBS) and after administration of heme using IF (Figure 1) and IHC (Supplementary Figure 1). No structural differences were detected in the kidneys or livers of heme-injected mice (Periodic Acid Schiff (PAS) and hematoxylin, eosin, saffron (HES) staining), in agreement with previous observations (13).

IHC staining for C3 activation fragments revealed minimal/absent staining in the three tested organs: kidney, heart and liver of PBS-injected mice. In heme-injected mice, significant deposits were observed in kidney glomeruli, while only minimal effects were observed in heart or liver using this technique (Supplementary Figures 1A–C). We also studied the C3 fragment deposition using the more sensitive IF method. C3 staining was detected in kidneys of PBS-injected mice, located along the tubular basement membrane and in the Bowman's capsule (Figure 1A); it was minimal/absent in heart, liver, brain, skin and lungs of these mice (Figures 1B–F). In the presence of heme, strong C3b/iC3b staining was detected in kidneys (Figure 1A, Supplementary Figure 1D) contrary to heart and brain (Figures 1B,D). To a lesser extent, deposits were also revealed in liver, skin and lungs (Figures 1C,E,F). In the heme-exposed kidneys themselves, C3 fragment deposits were observed in vessels, intra-glomeruli, along the tubular basement membrane and in the Bowman's capsule. C3 deposits were detected on the glomerular EC, as seen by double staining for C3b/iC3b and vWF (Supplementary Figure 1D).

### Long-Term Exposure to Heme Rendered Glomerular Endothelial Cells Susceptible to Complement Activation *in vitro*

**Figure 2:**
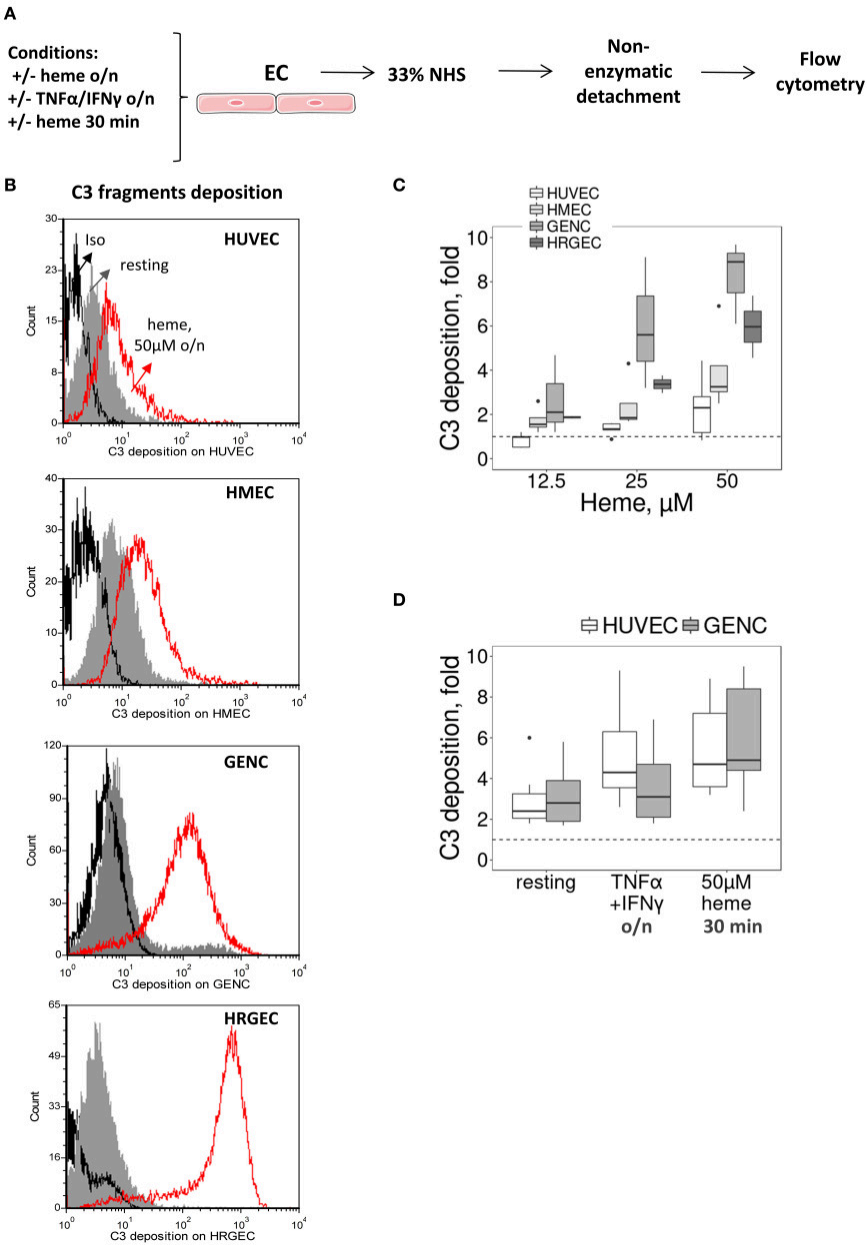
Influence of heme and cytokines on C3 fragment deposits on cultured human EC. **(A)** Experimental settings of figures **(B–D)** study by flow cytometry of C3b/iC3b deposition on cultured human EC (HUVEC, HMEC, GENC, HREGC), pretreated with medium only, TNFα/IFNγ overnight, or 50 μM of heme (30 min or overnight) before incubation with 33% NHS diluted in M199 culture medium and non-enzymatic detachment. **(B)** C3b/iC3b fragment deposits on resting EC (gray histograms) or EC treated overnight with 50 μM of heme (red histograms). Isotype control IgG1 appears as a black histogram (Iso). Representative histograms of 3 independent experiments. **(C)** Quantification of C3b/iC3b fragments deposition on the four EC types, studied as a function of the dose of heme overnight (*n* = 3–5). **(D)** Quantification of C3b/iC3b fragments deposition on HUVEC and GENC pretreated with medium only, TNFα/IFNγ overnight, or 50 μM of heme (30 min) (*n* = 3–5).

We then tested whether the particular susceptibility of the glomerular endothelium to C3 activation fragment deposition could be reproduced *in vitro*, using cultured human EC from different vascular beds (Figure 2A). We compared human primary (HRGEC) and conditionally immortalized (GENC) glomerular cells with HUVEC (as a macrovascular EC model) and human microvascular EC line (HMEC), for their capacity to activate complement after exposure to heme. In accordance with the *in vivo* data, C3 deposits were significantly higher on glomerular EC (GENC and HRGEC) than on HUVEC and HMEC (*p* < 0.05). Overnight exposure to 50μM heme increased C3 deposition on average ~2.6 fold on HUVEC, ~4 fold on HMEC, ~7.5 fold on GENC and ~6 fold on HRGEC compared with the baseline. No significant difference was observed in terms of C3 fragment deposition at resting state on the tested EC (Figures 2B,C).

Others TMA-related stimuli were tested, such as exposure to pro-inflammatory cytokines TNFα and IFNγ (deemed pertinent as the primary trigger for aHUS could be an infection) or brief exposure (30 min) to heme [since hemolysis has been proposed as a secondary hit for aHUS (8)]. In both cases HUVEC and GENC showed a similar response profile, with increased C3 fragment deposition from NHS, but without significant difference between the cell types in either the resting state or in the 30 min heme-exposed cells, while GENC had lower levels of C3 deposition compared to HUVEC after activation by the pro-inflammatory cytokines (*p* < 0.0001) (Figure 2D).

### The Increase of C3 Deposition on Glomerular EC Correlates With a Weaker FH Binding

**Figure 3:**
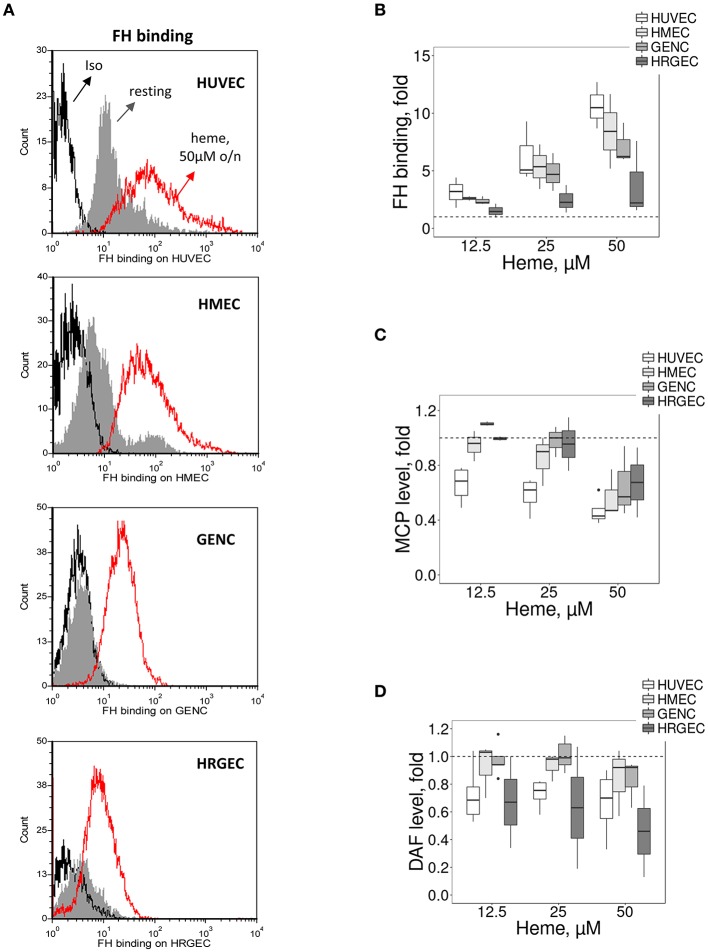
Influence of heme on C3 convertase regulators on cultured human EC. Experimental settings are similar with Figure 2. **(A)** Binding of FH on resting EC (gray histograms) or EC treated overnight with 50 μM of heme (red histograms) before incubation with sera. FH binding was analyzed by flow cytometry. Isotype control IgG1 appears as a black histogram (Iso). Representative histograms of 3 independent experiments. Quantification of the **(B)** FH, **(C)** MCP, and **(D)** DAF staining, studied as a function of the dose of heme (*n* = 3–5).

Most cells adapt over the long term to resist to heme overload. Therefore, we compared the binding/expression of complement regulators after overnight exposure to heme (Figure 3). We detected a heme-concentration-dependent increase of FH binding from NHS, significantly higher on HUVEC, compared with the glomerular EC (*p* < 0.05) (Figures 3A,B). The expression of MCP and DAF did not differ among the four tested EC types at resting state (Supplementary Figures 2A,B), while under hemolytic conditions MCP decreased to a similar level for the four cell types and DAF displayed a heterogeneous behavior (Figures 3C,D).

### TM Is Inefficiently Upregulated on Heme-Exposed Microvascular EC *in vivo* and *in vitro*

**Figure 4:**
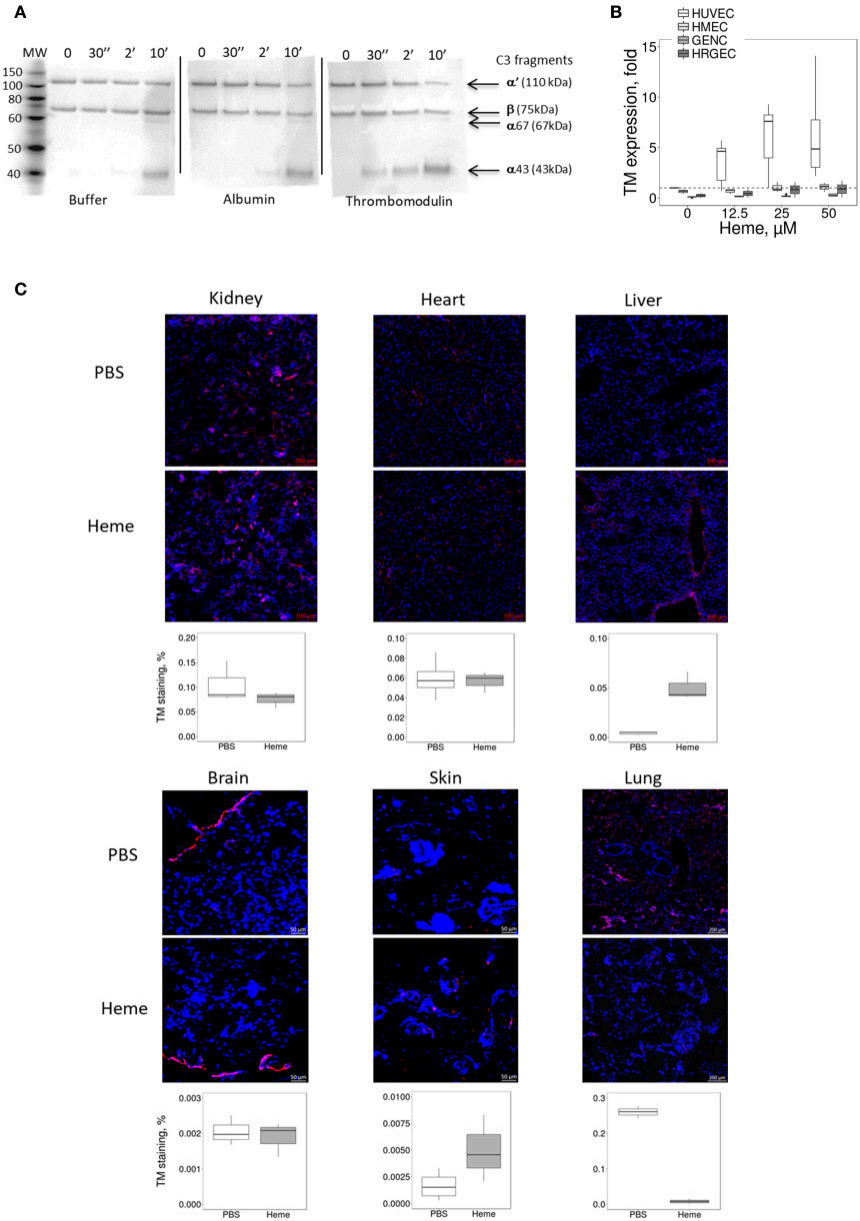
Influence of heme on the TM expression *in vitro* on cultured human EC and in mice. **(A)** Functional activity of TM as complement regulator, studied using purified protein by western blot. C3b is composed of 2 chains, α and β, visible by WB as 2 bands. The α chain is cleaved by FI to give two new bands: α67 and α43. The intensity of these bands reflects the level of C3b conversion to iC3b. Incubation of C3b with FH and FI led to cleavage of C3b to iC3b in a time-dependent manner. Addition of TM accelerates the cleavage of C3b compared to albumin and buffer controls. **(B,C)** HUVEC, HMEC, GENC, and HRGEC were incubated with increasing concentrations of heme overnight and mRNA was extracted. Gene expression of **(B)** TM was measured by RTqPCR, (*n* = 3). **(C)** TM staining (in red) was studied by IF in frozen kidney (x15), heart (x15), liver (x8), brain (x8), skin (x15), and lung (x5) sections of mice treated with PBS (negative control) or heme. Representative images of 5 mice per group. Lower panels: quantification of TM staining in organs sections of PBS (white) and heme-injected mice (gray).

The susceptibility of microvasculature to TMA could be related not only to lower resistance to complement but also to inefficient thromboresistance or regulation of the coagulation cascade. TM is a key factor in reducing blood coagulation, but may serve also as complement regulator (23). Here, TM accelerated the cleavage of C3b to iC3b by FI, leading to the appearance of the α43 fragment at 30 s with a time-dependent increase; this band was invisible in the presence of albumin (irrelevant protein) or buffer at 30 s, appearing later (2 min) with a time-dependent increase (Figure 4A).

*In vitro*, the expression of TM did not differ among the four tested EC types at resting state (Supplementary Figure 3). After prolonged exposure to heme, *TM* gene expression (Figure 4B) increased in HUVEC (gene expression ~6.1 fold at 50 μM heme, compared to baseline), while it was significantly lower in HMEC, GENC, and HRGEC (*p* < 0.05) compared with HUVEC. The TM level was also evaluated in different organs of heme-injected mice (Figure 4C). No difference was detected in glomeruli (strong basal staining) nor in heart or brain of heme- vs. PBS-injected mice. In contrast, a tendency toward an increase of TM was detected in skin, and a significant 18-fold increase of TM staining was found in the large vessels of the livers from heme-injected mice compared with PBS controls (*p* < 0.05). Surprisingly, an over 30-fold decrease of TM was seen in the lungs' microvasculature of heme-injected mice compared with controls (*p* < 0.0001) (Figure 4C).

### Heme Oxygenase-1 (HO-1) Is Inefficiently Upregulated on Microvascular and Particularly on Glomerular Endothelium by Heme *in vitro* and *in vivo*

The susceptibility of the kidney to TMA lesions could be also related to a reduced resistance to oxidative stress and inefficient heme-degradation. HO-1 is the major heme-degrading enzyme and its expression is inducible after cell stress (15).

**Figure 5:**
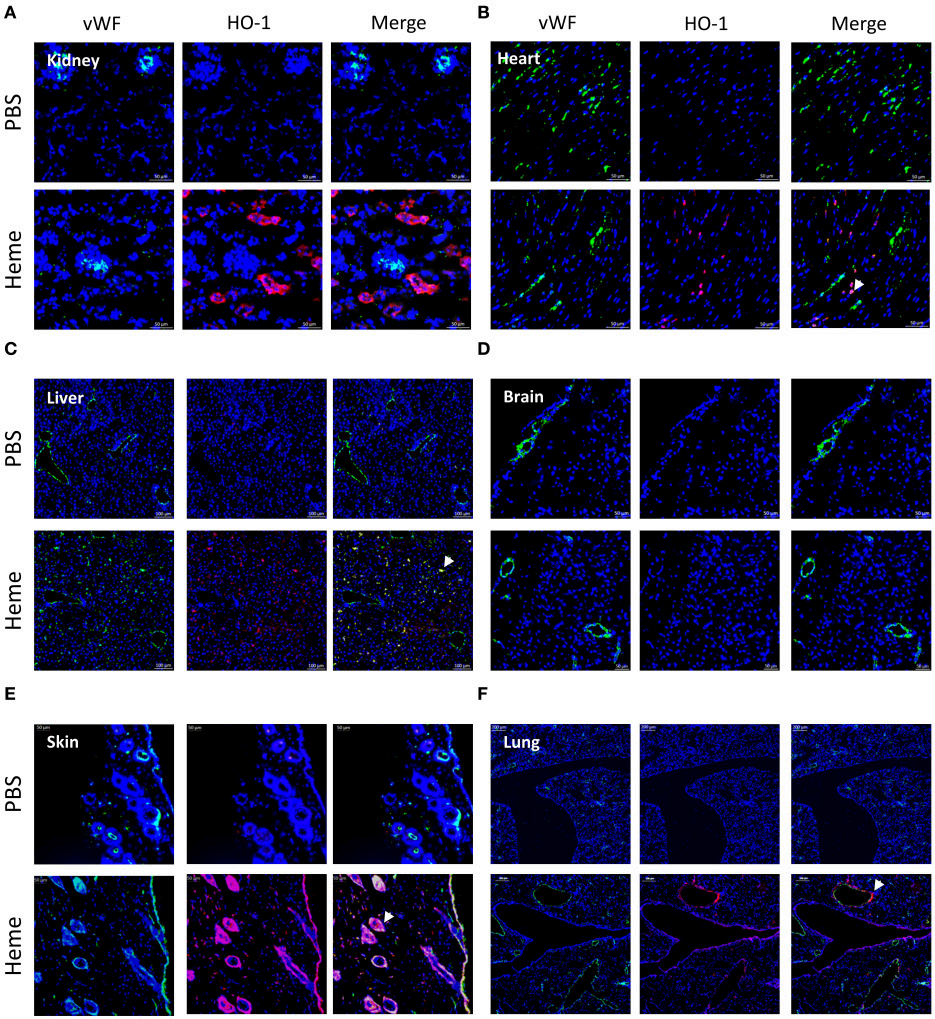
Comparison of HO-1 expression in different organs after heme injection in mice. **(A-F)** vWF (green) staining, HO-1 (red) staining and colocalization on frozen kidney (x15), heart (x15) liver (x8), brain (x8), skin (x15), and lung (x15) sections of mice, injected with PBS (upper panel) or heme (lower panel), studied by IF. Representative results of 3 independent experiments with 3 or 5 mice per group.

In mice, at basal condition (PBS), HO-1 staining was negative in the kidney (Figure 5A) as well as in others studied organs (Figures 5B–F). After heme injection, HO-1 staining was intense in the proximal tubules, but remained negative in glomeruli (Figure 5A, Supplementary Figures 4A,B). The same results were observed by IHC. Only minimal HO-1 staining was detected in some cases in heme-exposed glomeruli, corresponding to the glomerular epithelial cells and not endothelial cells in agreement with previous reports for human glomeruli (24); HO-1 expression was not detected in the brains of the heme-treated mice either. HO-1 labeling was positive in heart, liver, skin and lungs of heme-treated animals (Figures 5B–F), and visual colocalization with blood vessels was observed. In the skin, the HO-1 expression was particularly strong in the vessels of the hair follicles.

**Figure 6:**
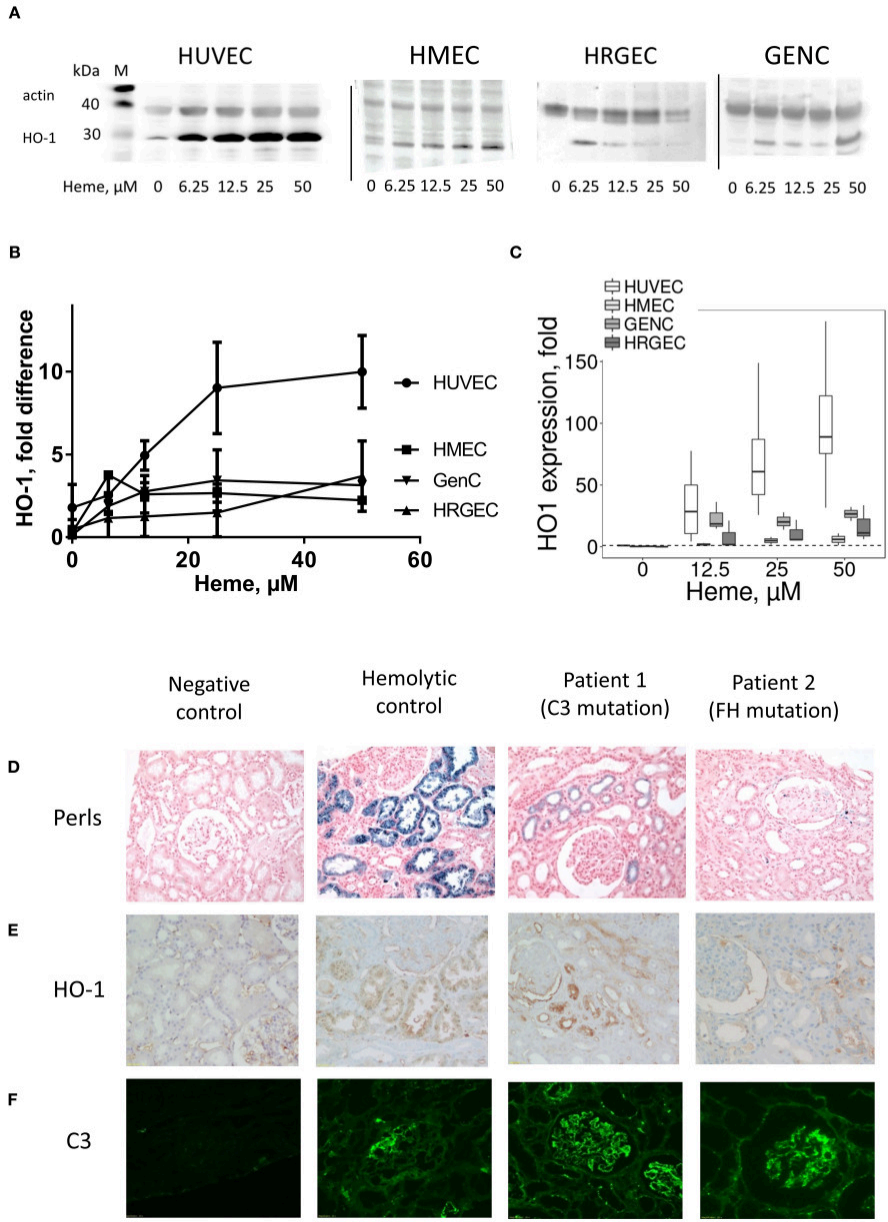
Influence of heme on the HO-1 expression *in vitro* in cultured human EC and HO-1 expression and C3b deposition in human kidney biopsies under hemolysis **(A–C)** HUVEC, HMEC, GENC, and HRGEC were stimulated with increasing concentrations of heme overnight. **(A)** HO-1 protein level studied by western blot (*n* = 2–5). **(B)** Quantification of the western blots (*n* = 2–5), ratio of the band of HO-1, relative to the band of actin. **(C)** *HO-1* gene expression measured by RTqPCR (*n* = 3). **(D)** Hemolysis level on human kidney biopsies was evaluated by the hemosiderin deposits, revealed by Perls' coloration. **(E)** HO-1 (brown) and **(F)** C3 (green) staining were performed by IHC and IF, respectively. A normal protocol, kidney allograft biopsy performed 3 months after transplant was used as a negative control. Biopsies of a patient with chronic hemolysis (hemolysis associated with prosthetic heart valve) were used as a positive control. Two patients with aHUS carrying complement mutations were tested.

*In vitro*, heme-treatment induced ~92.3 fold increased *HO-1* gene expression on HUVEC, compared to baseline (Figure 6C).This increase was weaker in the other cell types: ~5.9 fold on HMEC (*p* < 0.05), ~26.6 fold on GENC (*p* < 0.05) and ~17 fold on HRGEC (*p* < 0.05). HO-1 protein expression level in HUVEC, and to a lesser extent HMEC, increased with increasing heme concentration (Figures 6A,B—note that HMEC are derived from cells isolated from human foreskins and lack hair follicles). Meanwhile, HO-1 expression on both glomerular EC types was only weakly detected.

### HO-1 Is Inefficiently Upregulated in Patients' Glomerular Endothelium

In kidney biopsies of patients with hemolysis—aHUS or with prosthetic cardiac valve—IHC with anti-HO-1 revealed a tubular staining proportional to the degree of hemolysis observed by hemosiderin deposition by Perls' Prussian blue staining (Figure 6D). Glomerular endothelium HO-1 staining was negative (Figure 6E). No hemosiderin deposition, HO-1 mesangial or tubular stainings were detected in negative controls. It should be noted that all biopsies, even negative controls, displayed non-specific staining due to capture of the antibody by erythrocytes. Positive staining was nevertheless detected on podocytes, in agreement with previous observations (25). Similar to the mouse results, weak positive C3 staining was detected in some glomeruli of cardiac valve patients, while intense deposits were observed in the patients with complement abnormalities (Figure 6F).

### Glomerular EC Fail to Accommodate a Second Challenge With Heme and Show Enhanced C3 Deposition

**Figure 7:**
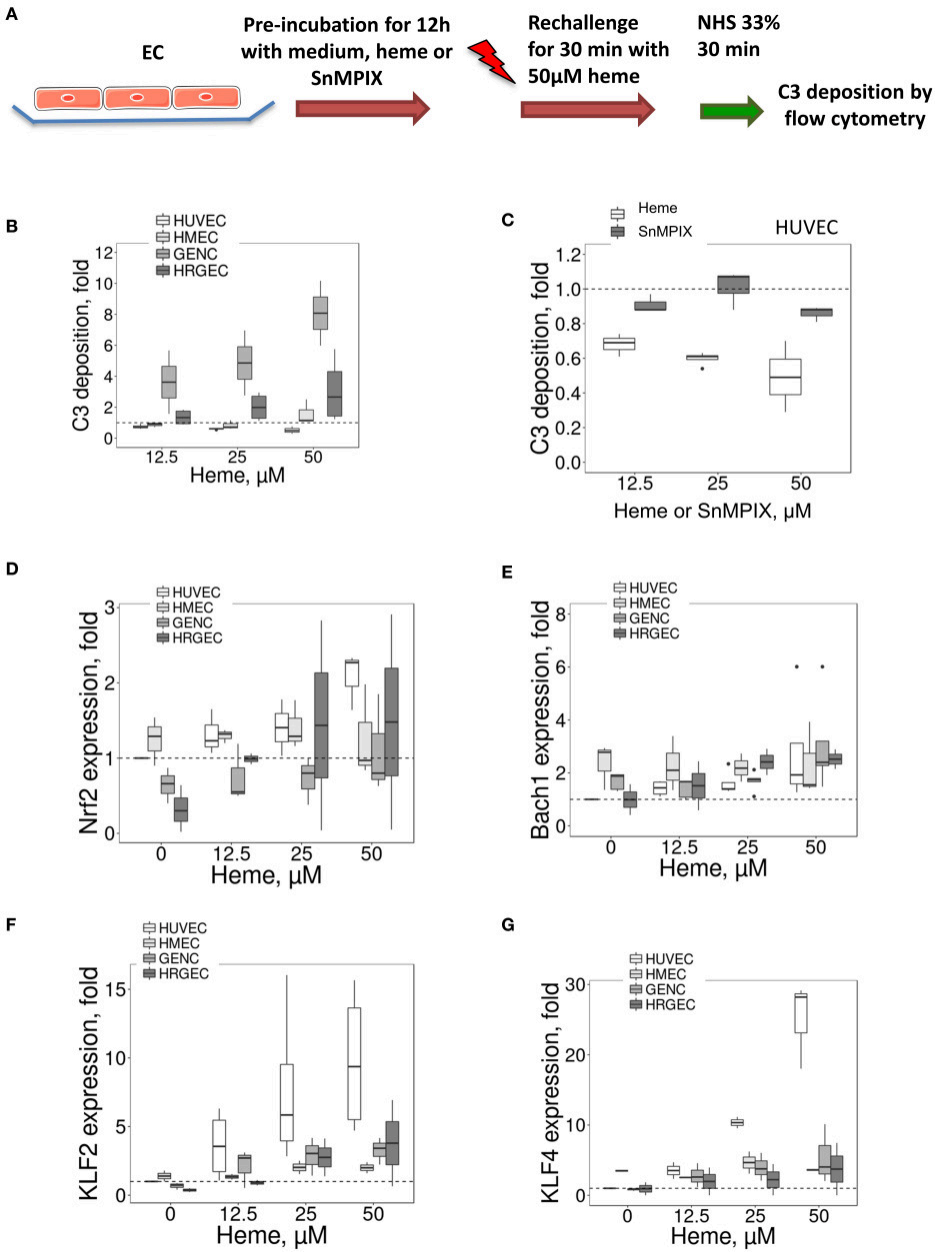
Influence of porphyrin pre-incubation on C3 fragments deposition on EC and expression of HO-1 and TM transcription factors **(A)** Experimental settings of **B** and **C**: C3 fragments deposition study, on cultured human EC pretreated overnight with medium only or pre-incubated with porphyrin (heme or SnMPIX) and then rechallenged by 50 μM of heme during 30 min, followed by incubation with 33% NHS. C3b/iC3b staining was analyzed by flow cytometry. **(B,C)** C3 fragments deposition under the experimental conditions described above as a function of the dose of heme during the pre-incubation step **(B)** on EC, and as a function of the dose of heme or SnMPIX **(C)** during the pre-incubation step on HUVEC (*n* = 3). **(D–G)** HUVEC, HMEC, GENC, and HRGEC were stimulated with increasing concentrations of heme overnight. **(D)** *NRF2*, **(E)** *BACH1*, **(F)** *KLF2*, and **(G)** *KLF4* gene expression measured by RTqPCR (*n* = 3).

The HO-1 up-regulation is accompanied by accommodation and a gain in complement resistance in HUVEC (16). We here compared the C3 deposition on the four EC types after incubation overnight with different doses of heme (to induce HO-1 upregulation), followed by a second challenge with 50 μM of heme for 30 min and exposure to serum (Figure 7A). The 30 min heme-exposure caused about a 2-fold increase of the C3 deposition on EC not receiving heme overnight (not shown). Exposure to heme overnight followed by 50 μM heme for 30 min produced a dose-dependent decrease in C3 deposition on HUVEC, but an increase on glomerular EC (Figure 7B). C3 deposits decreased by ~50% on HUVEC, while they significantly increased by ~8 fold on GENC and ~3 fold on HRGEC (*p* < 0.05) compared with HUVEC. HMEC showed an intermediate phenotype with C3 deposition comparable to baseline.

To find out to what extent this protective effect in HUVEC was HO-1 dependent, the HO-1 expression was induced by pre-incubation with another porphyrin of similar structure to heme (SnMPIX) but which blocks its activity (26–28). Overnight exposure of the cells to SnMPIX caused HO-1 expression, albeit 2–3 fold weaker than that induced by exposure to heme, and no increase of C3 deposition (data not shown). After a second challenge with heme, no protective effect against complement of the pre-incubation with SnMPIX was detected at any of the tested doses (up to 50 μM), contrary to heme which produced a protective effect after cells were exposed at 12.5 μM heme (*p* < 0.05) (Figure 7C).

### TM and HO-1 Expression May Be Parallel Phenomena, Dependent on the Transcription Activity of KLF2 and KLF4

HO-1 expression is mainly dependent of the transcription factors *NRF2* and *BACH1*, but no major differences were found for their expression between the four EC types (Figures 7D,E), except for a slightly lower expression of *NRF2* at resting state for the glomerular HRGEC (*p* < 0.05).

We then studied transcription factors *KLF2 and KLF4* because they control the expression of TM (29), and KLF4 could also modulate HO-1 expression (30). For *KLF2*, a strong dose-dependent effect was noted in HUVEC with ~9.8-fold increase compared with untreated cells after exposure to 50 μM of heme (Figure 7F). The increase in the other cell types was lower compared to HUVEC, with only ~2, ~3.3, and ~3.8-fold after exposure to 50 μM heme for HMEC, GENC, and HRGEC, respectively. Strong *KLF4* gene expression was also observed with increases in heme concentration (Figure 7G). Exposure to 50 μM heme induced ~25-fold increase of *KLF4* level in HUVEC, but only ~1.26, ~1.1, and ~1.48-fold in the microvascular HMEC, GENC, and HRGEC, respectively. These results may be explained, at least in part, by the shared profile of TM and HO-1 expressions in microvascular EC.

## Discussion

**Figure 8:**
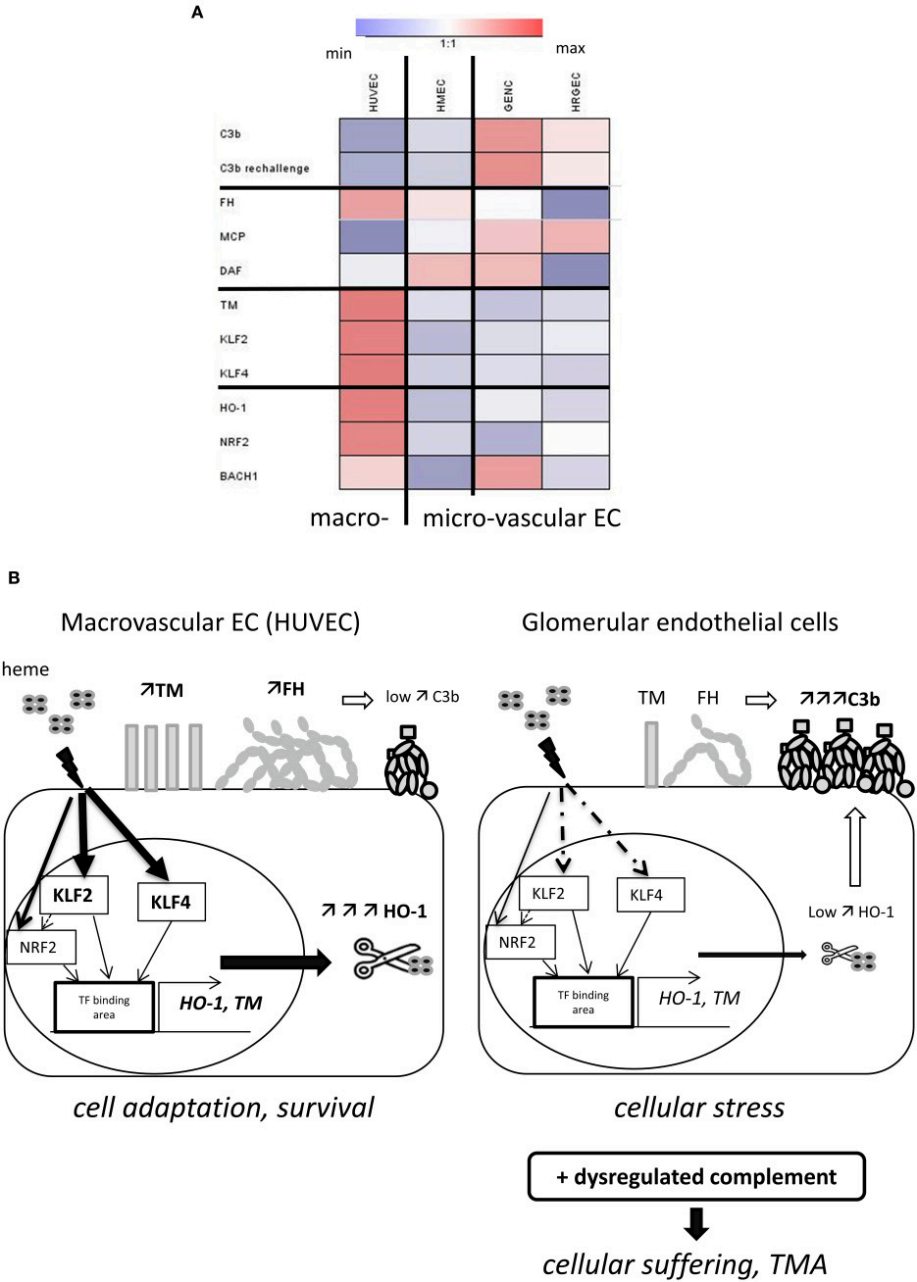
Schematic representation of our model to explain the glomerular vulnerability to complement-mediated TMA. **(A)** Heat map, illustrating the variations of the studied parameters between the four EC types. Results of the fold difference of gene or protein expression or binding with exposure to 50μM heme were analyzed using Genesis software. White color indicates no difference (fold change 1), blue indicates reduction and red an increased expression/binding. **(B)** Proposed model to explain the microvascular/glomerular cell susceptibility of TMA lesions. Our results suggest that hemolysis is a key factor in the vulnerability of the glomerular EC complement overactivation and TMA. The glomerular EC have a weaker capacity to adapt to hemolysis and to up-regulate cytoprotective and stress-response genes, such as HO-1 and TM, compared to EC from other organs. This could be related to the lower levels of the transcription factors *KLF2* and *KLF4*. The inefficient degradation of heme due to the lack of HO-1 will result in stronger heme-mediated complement activation on the endothelium. The weaker expression of TM will on the one hand contribute to the pro-coagulant phenotype of the EC, and on the other hand contribute to inefficient degradation of the anaphylatoxins C3a and C5a and assist FH and FI in degradation of C3b. Finally, FH will bind less to heme-exposed glomerular EC compared to EC of other organs. This will be further exacerbated in case of complement overactivation, as in aHUS, leading to EC suffering and TMA.

In this study, we discovered a dichotomy in the phenotypic adaptation to hemolysis of macro- vs. microvascular endothelium. Macrovascular EC adapted to heme-overload by up-regulating the heme-degrading HO-1 and the coagulation and complement regulator TM, as well as exhibiting enhanced binding of the complement regulator FH. On the contrary, the microvascular EC, and in particular glomerular EC, were less efficient at each of these processes (Figure 8A), which resulted in a susceptibility of glomerular endothelium to heme-driven complement overactivation. These observations may help to explain the microvascular and renal tropism of the complement-mediated TMA lesions of aHUS (Figure 8B).

The tropism of TMA lesions for small vessels is a defining feature of this group of syndromes, but the mechanisms remain poorly described. Our results and data from the literature suggest that resting microvascular EC express similar or even higher levels of complement regulators and TM (6, 31, 32), failing thus to explain their susceptibility to TMA lesions. It is well-established that the presence of shiga toxin in typical HUS, complement abnormalities in aHUS, or ADAMTS13 deficiency in thrombotic thrombocytopenic purpura (TTP) are necessary but not sufficient in themselves to trigger TMA (1, 33). Inflammatory insult is a frequently described TMA-trigger. Nevertheless, our results showed that inflammatory mediators increased complement activation and decreased TM expression in both micro- and macrovasular EC, in accordance with previous studies (6, 33–35). Secondary hits are needed to overcome the tolerable EC stress and to promote overt endothelial injury and disease manifestation. Since hemolysis is a hallmark of TMA, it is tempting to speculate that oxidative stress and cell activation will be particularly noxious for the microvessels, in the kidney as well as other organs, amplifying cell damage, complement activation and thrombosis, contrary to macrovessels.

Hemolysis alone also does not induce renal TMA lesions (13) but may present a secondary challenge in the presence of deregulated complement, as in the case of aHUS (7, 8); indeed, heme activates complement AP directly in serum and this contributes to the C3 deposits found on EC (8, 11, 36–39). In support of this hypothesis, injection of heme resulted in a marked renal C3 deposition in mice, localized predominantly in the glomeruli, and similar to what has been observed for intravascular hemolysis and injection of cell-free hemoglobin (39). In contrast to kidneys, our examination of the heart, liver, brain, skin and lungs showed them to be largely unaffected by injection of heme, or to have exhibited only small increases in C3 deposits after injection (summary in the Supplementary Table 1). In agreement with these *in vivo* data, overnight exposure to heme induced stronger C3 deposition on glomerular EC compared with the macrovascular (HUVEC) and microvascular (HMEC) cell models. Together, these *in vivo* and *in vitro* observations point toward a vulnerability of the glomerular endothelium to complement deposition in the presence of heme. However, the inefficient adaptation to hemolysis in terms of HO-1 expression and TM up-regulation was also observed in other microvascular EC, both *in vitro* and *in vivo*, while only glomerular EC were subject to deposition of very high levels of C3 activation fragments upon exposure to heme.

These particularly elevated complement deposits in kidneys and glomerular cells may be explained by reduced binding of FH, the main regulator of the AP of complement (3), which is the most frequently affected among aHUS patients [genetic or acquired abnormalities found in >40% of patients (2, 40, 41)]. Moreover, we confirmed that FH is assisted by TM in the inactivation of C3b (23, 42, 43) and that the lower level of TM can further aggravate the inefficient complement regulation. The underlying mechanism(s) behind this reduced FH binding require further investigation, but the combination of decreased FH binding to glomerular EC under hemolytic conditions with FH mutations or autoantibodies could contribute to the susceptibility of glomerular EC to TMA lesions. Interestingly, a recent study also showed that renin, an enzyme produced specifically in the kidney, could cleave C3 and exacerbate the complement activation (44). The hemolysis-mediated complement activation could be an additional key element in this vicious circle, linking renin secretion, vascular damage and renal failure. Thus, the C3 deposits described in aHUS kidney biopsies, which are generally considered non-specific (45, 46), could reflect the complement activation due to hemolysis and deregulated complement, this last condition explaining why the C3 deposits are greater in aHUS patients than in cases of isolated hemolysis (cardiac valve).

The cells' capacity to express HO-1, to degrade heme and then detoxify it to cytoprotective metabolites biliverdin and CO, plays a key role in vascular protection (15, 47). *In vitro* studies have shown heme-mediated HO-1 expression in HUVEC (48, 49), and there is clear evidence for endothelial HO-1 expression in large vessels of patients with sickle cell disease (50), as well as in animal models of hemolysis or other stress inducers (51–55). In contrast, we found only HO-1 upregulation to be minimal or absent in glomerular EC (*in vitro* or *in vivo*), in aHUS and hemolytic patients, as well as in heme-injected mice. This observation is in agreement with published images of aHUS and under other hemolytic conditions (20, 24, 25, 56, 57) and indicates that glomerular endothelium does not have the capacity to efficiently up-regulate HO-1 in presence of heme. HO-1 production is, however, indispensable to the glomerular protection, as suggested by reported early damages of glomeruli in case of HO-1deficiency in murine model or in human (47, 58, 59). Mesangial proliferation and thickening of the capillary wall in accordance with endothelial swelling and detachment were indeed described in a HO-1 deficient young boy. This early aspect of mesangioproliferative glomerulonephritis was confirmed from sequential kidney samples (at 2, 5 and 6 year-old), while tubulo-interstitial damages advanced more progressively. This could to be related to the fact that the production of HO-1 within kidneys would be mainly tubular, while in the glomeruli the major HO-1 source would be the infiltrating macrophages, not the intrinsic glomerular cells (60). Further studies are needed to extend our knowledge on the different ways, that the EC from different vascular beds manage heme homeostasis, based on their specific ability to accumulate it, to transport it within the cell by FLVCR1, to express ferritin, store iron and to produce reactive oxygen species. Some of these parameters have been studied for HUVEC and HMEC, especially in the context of deficiency of the heme transporter FLVCR1a, but data on glomerular EC are lacking (61). Moreover, a recent study in the context of leukemia demonstrated that C3a and C5a trigger phosphorylation of MAPK, followed by downregulation of HO-1 expression in malignant cells (62). If such phenomenon operates in HO-1 expressing endothelium, complement activation by intravascular hemolysis and heme release may weaken the endothelial resistance. In such context, inhibitors of MAPK (such as SB203580) will result in upregulation of HO-1 and enhance resistance to hemolysis-derived products as well as to complement.

A protective role of HO-1 against immune complexes-mediated complement overactivation has also been described on HUVEC (16). Here, our results demonstrated a complement-protective effect of pre-incubation with heme in HUVEC. Ideally, this phenomenon should be confirmed after HO-1 silencing. In contrast, the glomerular EC showed a marked increase in C3 deposition. These results, together with the loss of this protective effect upon HO-1 inhibition in HUVEC, suggest that HO-1 activity for heme degradation contributes to protection against complement activation in this cell type—a phenomenon which is not operational in the glomerular endothelium. It is important to note that glomerular EC were able to upregulate HO-1 after incubation with a very potent inducer, such as CoPPIX (data not shown), suggesting that the machinery needed for its synthesis is present and can be triggered after potent stimulation. Nevertheless, induction of massive hemolysis *in vivo* was not strong enough to mediate HO-1 staining in glomerular EC, contrary to tubuli and podocytes [(13) and data not shown]. Our *in vivo* experiments also showed that the brain endothelium failed to upregulate HO-1 after heme injection, but this was not associated with concomitant deposition of C3 fragments. Sartain et al. recently demonstrated that brain microvascular EC express higher levels of complement regulators, compared to glomerular EC, and had a better capacity for suppressing the alternative pathway *in vitro* (32). These findings may help to explain the occurrence of lower levels of cerebral vessels' complement deposits and the fact that in aHUS, as well as in shiga toxin HUS, brain manifestations were found in only a small fraction of patients (63). On the other hand, the inefficient upregulation of HO-1 in the brain vasculature may contribute to the cerebral manifestations of TTP (also a TMA disease). The possible role of HO-1 expression in TTP pathophysiology requires further studies.

Interestingly, *KLF2* (64, 65), and *KLF4* (66), which are known transcription factors of TM, have also been shown to serve as modulators of *HO-1* expression (30). Therefore, the weaker up-regulation of TM and HO-1 we observed in microvascular EC could at least in part be related to differences in transcription regulation. Consistent with these data, other workers have reported that *KLF2* and *KLF4* were not significantly upregulated in glomeruli of aHUS patients compared with controls (67).

The observed differences in gene expression and phenotype among the tested EC here confirm the utility of HUVEC and HMEC as model EC types for macro- and microvascular endothelial, but highlight unique features and responses to activation in glomerular EC, which could perhaps be better modeled by glomerular (primary or cell line) EC in culture. A limitation of our work is that, although we observed correlations, we do not provide direct evidence linking HO-1 and complement deposits *in vitro*, for which the knock out/knock in strategy would have been useful. Also, providing *in vivo* evidence that up-regulation of HO-1 could prevent complement activation and TMA lesions in a mouse model of aHUS was outside the scope of this study.

In conclusion, we have shown that when compared with macrovascular EC, microvascular EC, and glomerular EC in particular, are vulnerable to complement-mediated TMA at least in part because of their failure to adapt to hemolysis, to up-regulate cytoprotective and stress-response genes such as HO-1 and TM, and to bind FH (Figure 8B). We hypothesize that local, subclinical microthrombosis due to a primary triggering event (infection, pregnancy) will cause mechanical hemolysis in the kidney. By amplifying complement activation and pro-thrombogenic traits, heme will exacerbate endothelial activation. Once the threshold of tolerance is reached, this heme-induced endothelial and complement overactivation could sustain and perpetuate the TMA lesions. Together, our results indicate that the vulnerability of the glomerular EC to hemolysis is a key factor, predisposing them to complement overactivation and TMA, as seen in aHUS. The heme scavenger protein hemopexin has been efficient in preventing hemolysis-mediated C3 deposition in kidneys in a mouse model of intravascular hemolysis and on endothelial cells *in vitro* (39), as well as in models of stasis in sickle cell disease (68) or other hemolytic conditions (69). Therefore, heme-blocking agents may be explored as novel therapeutic strategies to prevent microvascular injury in TMA diseases.

## Author Contributions

LR, MF, and OM designed the study. OM, NM, AG, CP, TR-R, RP, VG, and FD performed research. SS and PM provided the GENC cell line. MH performed statistical analyses. LR, MF, OM, VF-B, JD, NM, AG, VG, and EB discussed the data. All authors wrote the manuscript and approved the submission.

### Conflict of Interest Statement

The authors declare that the research was conducted in the absence of any commercial or financial relationships that could be construed as a potential conflict of interest.
