# Supplementary material for: Heme Drives Susceptibility of Glomerular Endothelium to Complement Overactivation Due to Inefficient Upregulation of Heme Oxygenase-1
Source: Front Immunol. 2018 Dec 20;9:3008. doi: 10.3389/fimmu.2018.03008 (PMC6306430; doi:10.3389/fimmu.2018.03008)
Supplement: Supplementary file 1 [file Data_Sheet_1.PDF]

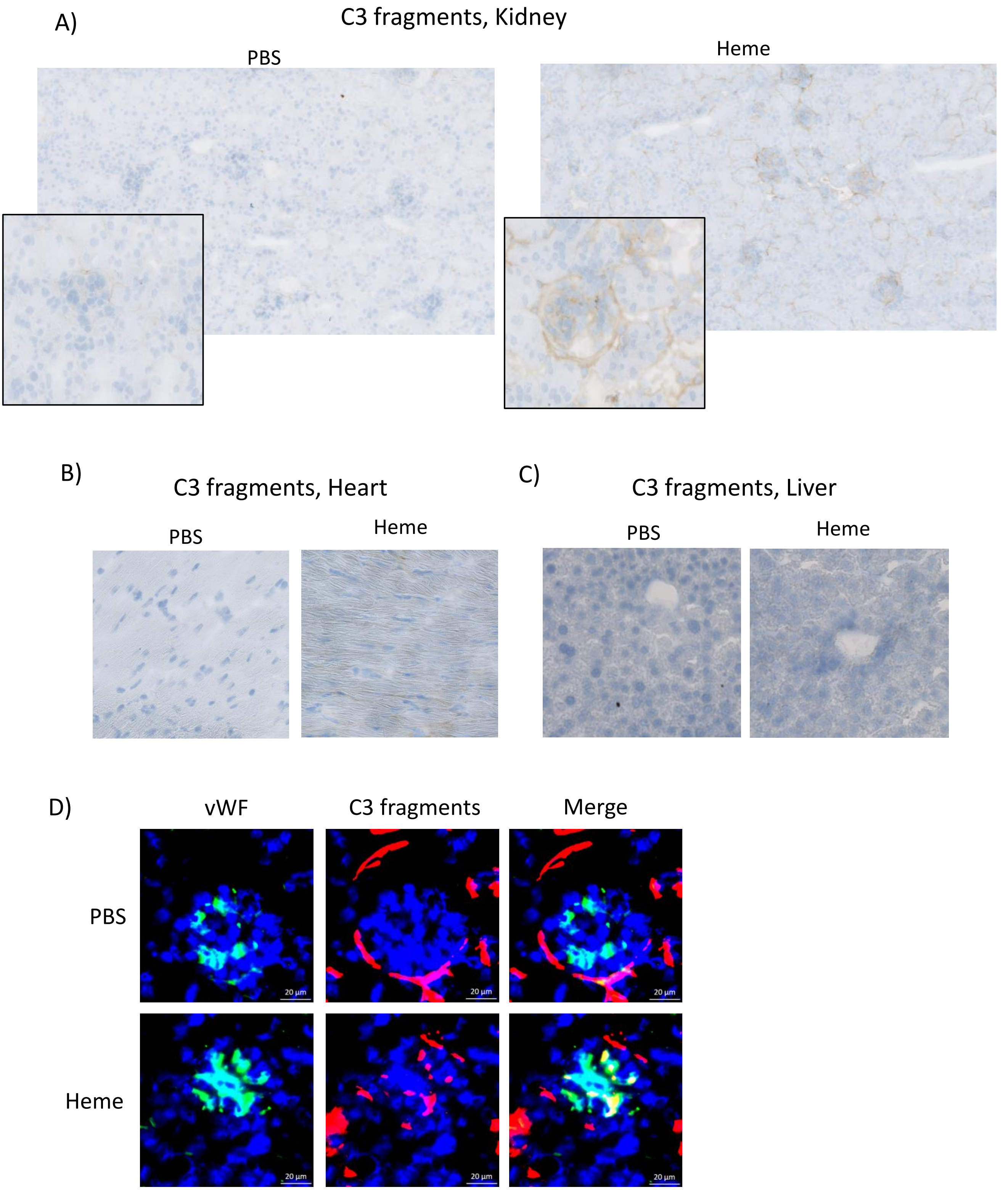

Supplementary Figure 1: Heme-induced complement C3 fragments deposition with a particular renal tropism *in vivo*. Comparison with another method. C3 fragment staining appears in brown on frozen kidneys (A), heart (B) and liver (C) sections of mice treated with PBS or heme, studied by immunohistochemistry. D) Focus on one frozen kidney glomerulus stained for vWF (green) and C3 fragments (red) (zoom x30).

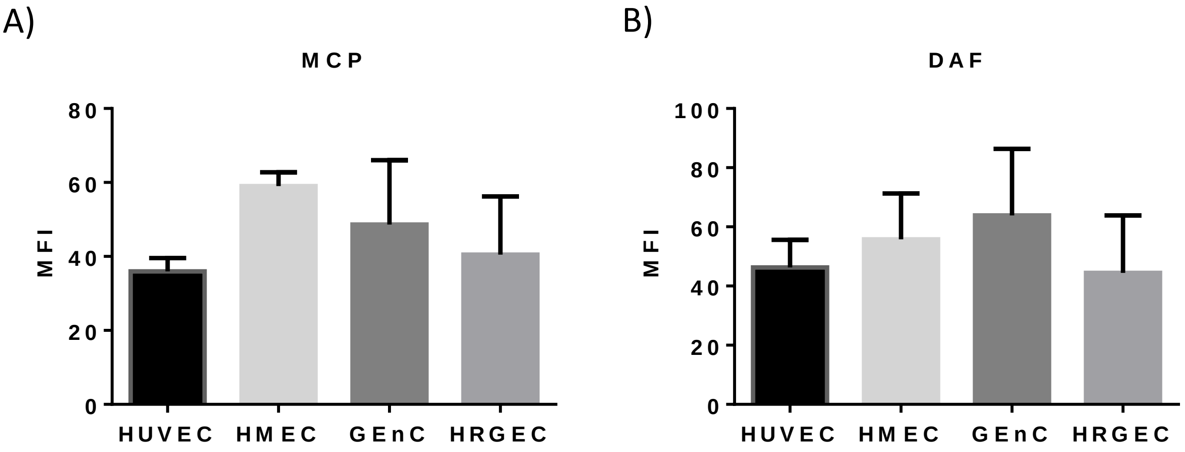

Supplementary Figure 2: Comparison of the expression of MCP and DAF on resting EC. Basal levels of expression of MCP (A) and DAF (B) were measured on resting HUVEC, HMEC, GEnC and HRGEC by flow cytometry.

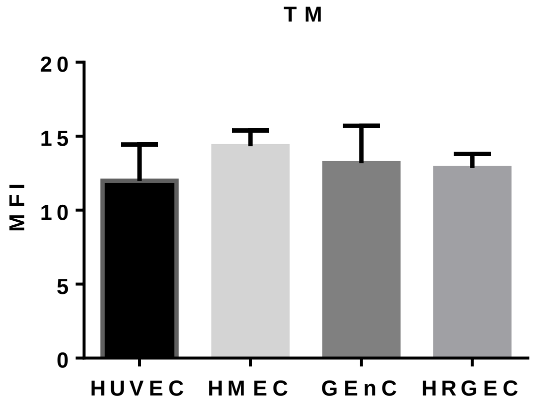

Supplementary Figure 3: Comparison of the expression of TM on resting EC. Basal level of expression of TM was measured on resting HUVEC, HMEC, GEnC and HRGEC by flow cytometry.

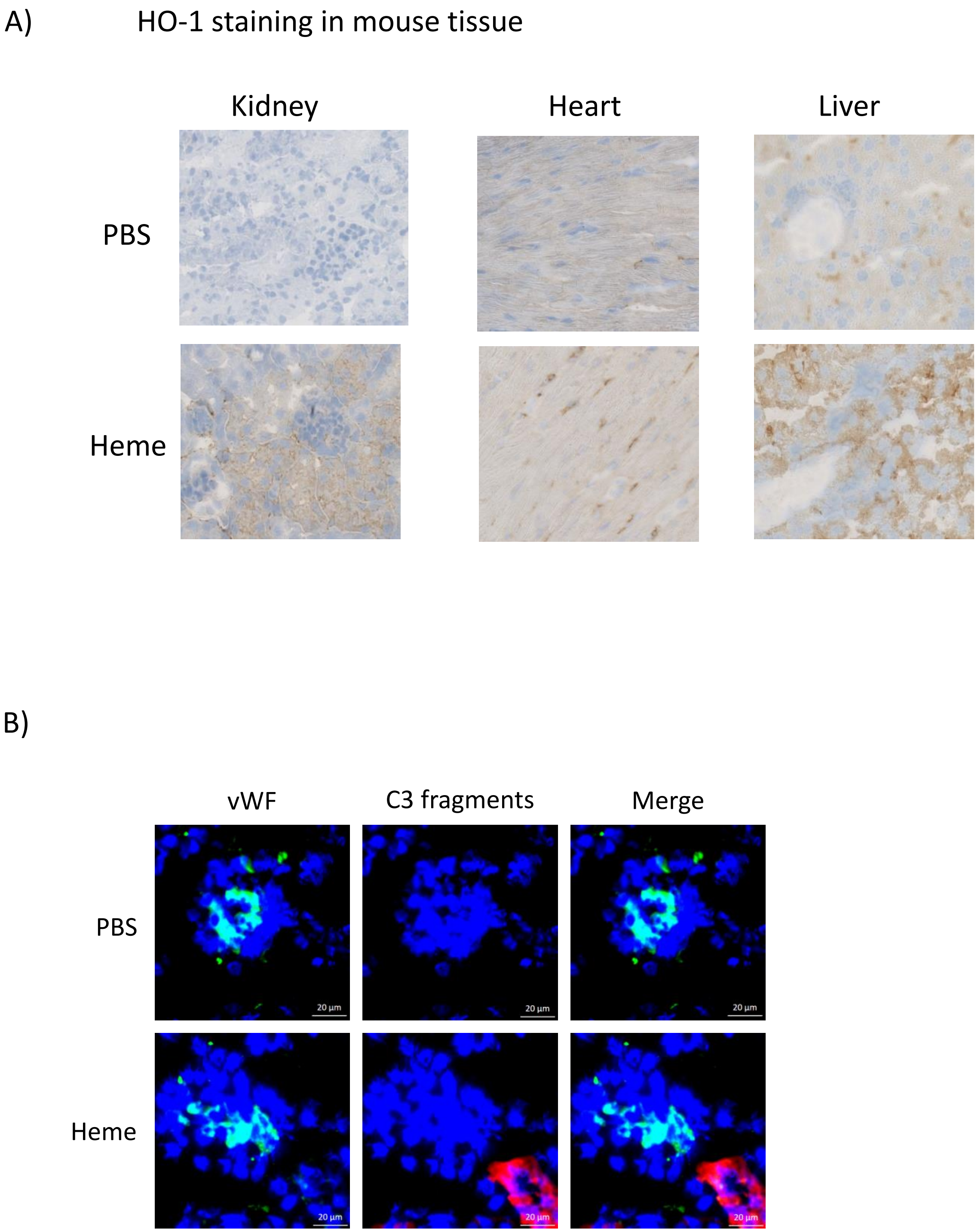

Supplementary Figure 4: Comparison of HO-1 expression in different organs after heme injection in mice with another method. A) HO-1 staining appears in brown on frozen kidneys, heart and liver sections of mice treated with PBS or heme, studied by immunohistochemistry. B) Focus on one frozen kidney glomerulus stained for vWF (green) and HO-1 (red) (zoom x30).

Heme-treatment	C3	HO-1	TM
Kidney glomeruli	↗↗↗	=	=
Liver	↗	↗↗	↗↗
Heart	↗	↗↗	=
Brain	=	=	=
Skin	=	↗↗↗	↗
Lungs	↗	↗↗↗	↘↘

Supplementary table 1. Summary of the staining in different organs
